# Enhanced optical output power of blue light-emitting diodes with quasi-aligned gold nanoparticles

**DOI:** 10.1186/1556-276X-9-7

**Published:** 2014-01-06

**Authors:** Yuanhao Jin, Qunqing Li, Guanhong Li, Mo Chen, Junku Liu, Yuan Zou, Kaili Jiang, Shoushan Fan

**Affiliations:** 1 State Key Laboratory of Low-Dimensional Quantum Physics, Department of Physics & Tsinghua-Foxconn Nanotechnology Research Center, Tsinghua University, Beijing 100084, China; 2 Collaborative Innovation Center of Quantum Matter, Beijing 100084, China

**Keywords:** GaN, Light-emitting diodes (LEDs), Au nanoparticles, Carbon nanotube

## Abstract

The output power of the light from GaN-based light-emitting diodes (LEDs) was enhanced by fabricating gold (Au) nanoparticles on the surface of p-GaN. Quasi-aligned Au nanoparticle arrays were prepared by depositing Au thin film on an aligned suspended carbon nanotube thin film surface and then putting the Au-CNT system on the surface of p-GaN and thermally annealing the sample. The size and position of the Au nanoparticles were confined by the carbon nanotube framework, and no other additional residual Au was distributed on the surface of the p-GaN substrate. The output power of the light from the LEDs with Au nanoparticles was enhanced by 55.3% for an injected current of 100 mA with the electrical property unchanged compared with the conventional planar LEDs. The enhancement may originate from the surface plasmon effect and scattering effect of the Au nanoparticles.

## Background

Much research has been devoted towards gallium nitride (GaN)-based semiconductor devices, especially in terms of applications for light-emitting diodes (LEDs), which operate in the blue and green wavelength regions. GaN-based LEDs have attracted interest for use in full-color display panels and solid-state lighting [1] because they have advantages such as low energy consumption, long lifetimes, and relatively small sizes. However, in conventional planar LEDs, the light extraction efficiency is limited by several factors including the high refractive index of p-GaN (approximately 2.52), leading to a low total internal reflection (TIR) angle [2]. To enhance the output light power, various approaches, such as surface texturing [3,4], photonic crystals [5-7], and metal oxide nanoparticles [8-11], have been studied.

Surface plasmons (excitations on a rough metallic surface by the interaction between light and the metal nanoparticles) were suggested as a way to significantly enhance the light emission efficiency in LEDs [12]. Several methods have been suggested to fabricate metal nanoparticles (NPs) on LEDs to improve their efficiency. These include thermal annealing process [13-16], chemical synthesis [17], and gas etching technique. For noble metal nanoparticles on GaN surfaces, the collective oscillations of the electrons are localized surface plasmons (LSPs) [18,19]. Under the resonance condition, this LSP effect enables the metallic nanoparticles to capture the trapped light in the p-GaN layer of the LEDs, enhancing the extracted efficiency of the light. However, the LSP resonance effect is affected by the geometry and separation of the nanoparticles. When noble metal nanoparticles are fabricated with a thermal annealing process, it is important to control the distribution and size of the nanoparticles. Furthermore, the residual metal after the annealing process has a negative influence on the device performance.

We report a simple method for making quasi-aligned Au nanoparticle arrays on p-GaN surfaces using superaligned multiwall carbon nanotubes (CNTs). The LED devices containing quasi-aligned Au particles exhibited efficient electrical properties, and the optical output power was significantly increased compared with devices without Au particles. By eliminating any chemical or etching processes, this method has potential for excellent integration with semiconductor technologies. Furthermore, we observed that the quasi-aligned Au nanoparticle arrays also had an effect on the polarization performance of the LEDs.

## Methods

**Figure 1:**
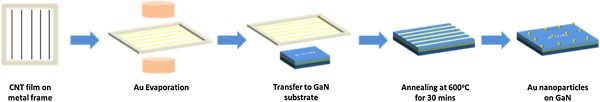
Fabrication process of the Au nanoparticles using an Au-CNT system.

The CNT thin films were directly drawn out from the CNT arrays [20], which were composed of CNTs with diameters around 10 nm and were aligned parallel in one direction. This method is convenient for mass production of CNT films at a low cost. In our experiment, the CNT thin films were pulled out from a superaligned CNT array grown on a 4-in.silicon wafer and fixed to metal frames. We then fabricated the Au films using electron beam evaporation on the suspended CNT films with thicknesses in the range of 1 to 5 nm. The GaN LED wafers consisted of a 200-nm-thick p-type GaN layer, a layer containing InGaN/GaN quantum wells, an n-type GaN layer, and a GaN buffer layer. The as-prepared Au-CNT films were transferred directly onto the GaN substrates. We used alcohol on the Au-CNT/GaN interface to make the carbon nanotubes shrink, allowing the film to form a close contact with the substrate. Afterwards, the Au-CNT films were thermally annealed at 600°C for 30 min in ambient air, and then the CNT films were completely removed because of the high temperature, inhibiting a decrease in the transmittance of the carbon nanotubes. During the annealing process, the metal Au films in the Au-CNT system formed Au nanoparticles that were bound to the surface. The fabrication process of the Au nanoparticles using an Au-CNT system is illustrated in Figure 1.

**Figure 2:**
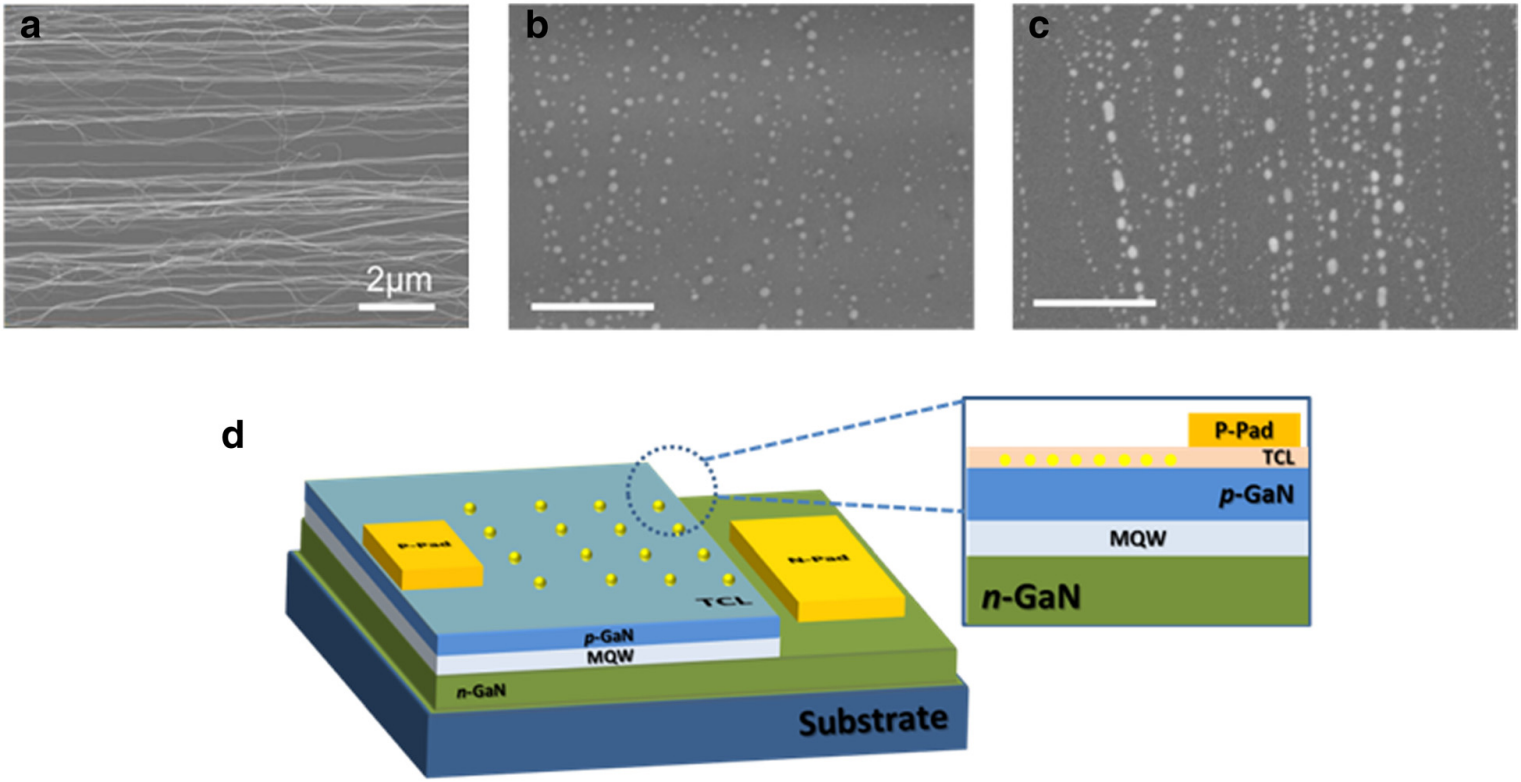
**SEM images and schematic 3D representation. (a)** SEM image of a carbon nanotube thin film (the scale bar is 2 μm). SEM images of Au nanoparticles from a **(b)** 2-nm and **(c)** 5-nm Au-CNT system where the scale bars are 500 nm. **(d)** Schematic 3D representation of a GaN LED with embedded Au nanoparticles.

A scanning electron microscope (SEM) image of a carbon nanotube thin film is shown in Figure 2a. Figure 2b,c shows top views of the scanning electron microscope images of the Au nanoparticles on GaN substrates that were derived from the 2- and 5-nm Au-CNT systems through an annealing process. The schematic representation of a GaN LED with embedded Au nanoparticles is shown in Figure 2d with a cross-sectional view of the local region. From Figure 2, it can be seen that the Au nanoparticles distributed along the former CNT path and the quasi-aligned particle arrays were formed. The CNT films played an important role in acting as a frame and could be easily removed with an annealing process. The Au was deposited around the CNTs, and there was no redundant Au deposited on the device surface. Thus, there was no residual Au that needed to be removed after the annealing process, preventing any negative impacts on the performance of the device from the optical and electrical aspect. Furthermore, we could control the distribution of the nanoparticles by adjusting the deposition volume. The size and density of the Au nanoparticles depended on the thickness of the Au film evaporated on the CNTs. Larger particles and a higher density distribution could be fabricated from the Au-CNT system with a thicker Au film. The size of the particles and their quantity changed continuously with the Au thickness on the CNT films. Figure 2 shows a comparison of the 2- and 5-nm Au-CNT systems to investigate the morphology of the Au nanoparticles obtained. Compared with the Au nanoparticles derived from the 2-nm Au-CNT system, the Au nanoparticles derived from the 5-nm Au-CNT system were larger in both size and quantity. The average diameters were around 20 to 25 nm and 30 to 35 nm for the nanoparticles derived from the 2- and 5-nm Au-CNT systems, respectively. The heights were measured using an atomic force microscope (AFM) acquired with a Veeco Dimension V (Veeco Instruments Inc., Plainview, NY, USA). The spaces between the nanoparticles were from 20 to 70 nm for the 2-nm Au-CNT system. The spaces between the nanoparticles from the 5-nm Au-CNT system were around 30 to 70 nm.

After fabricating the Au nanoparticles, the GaN wafers were used to fabricate LEDs using standard procedures with a mesa area of 1 mm^2^. A transparent conducting layer (TCL) of Ni (2 nm)/Au (5 nm) was deposited on the p-GaN surface. Ni (5 nm)/Au (100 nm) electrodes were then deposited by photolithography exposure and electron-beam evaporation on the n-GaN layer and the TCL as n- and p-pads, respectively. For comparison, a standard LED device was fabricated with a TCL deposited directly on the p-GaN surface with all other fabrication processes kept the same as those used for the Au nanoparticle LEDs.

## Results and discussion

**Figure 3:**
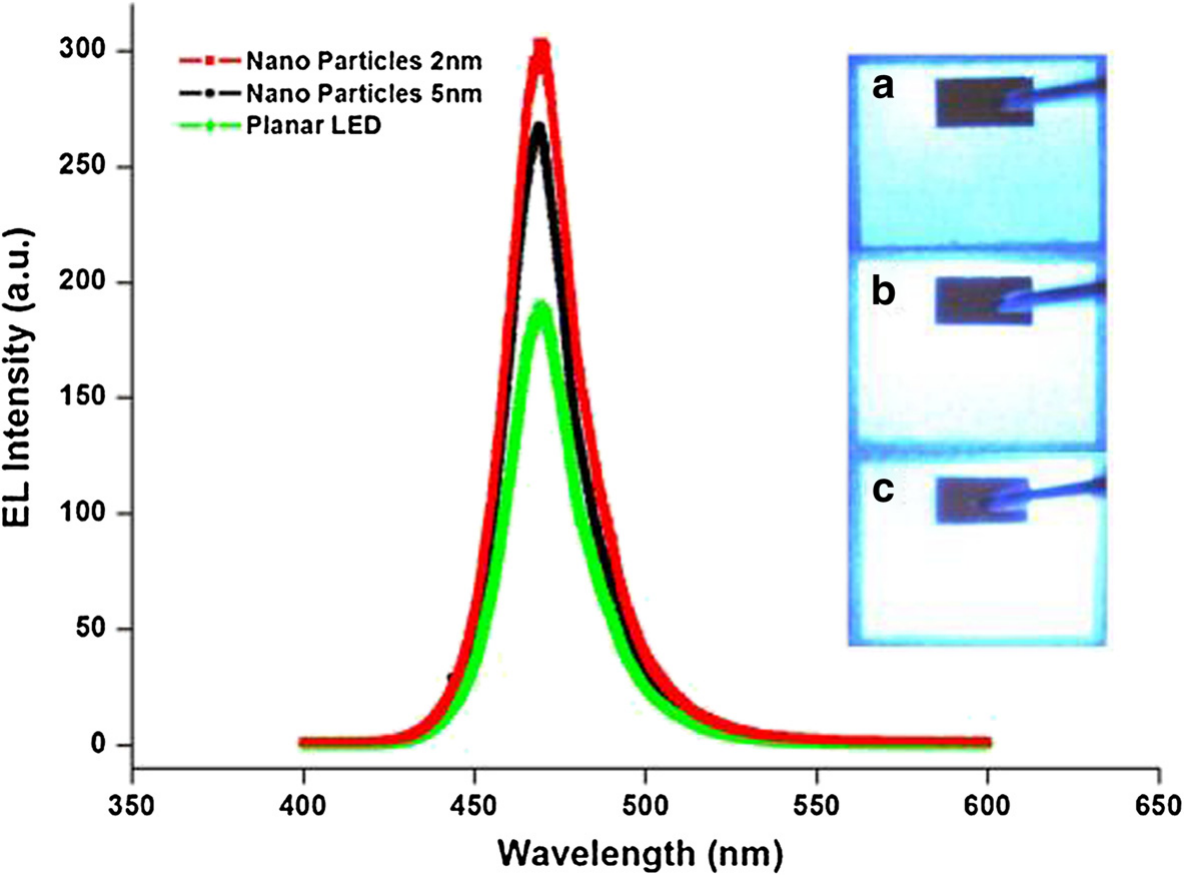
**EL spectra of LEDs.** The LEDs are with Au nanoparticles from the 2- and 5-nm Au-CNT systems with an injection current of 100 mA measured at room temperature, using a planar LED as a reference. The inset shows optical microscope images of the LEDs **(a)** without any Au nanoparticles, **(b)** with Au nanoparticles from the 5-nm Au-CNT system, and **(c)** with Au nanoparticles from the 2-nm Au-CNT system. All of the devices were operated with an injection current of 100 mA.

To evaluate the optical properties of the as-prepared LEDs, we performed electroluminescence (EL) spectroscopy experiments for all of the devices. The EL spectroscopy was measured from the top of the samples with forward injection currents from 10 to 100 mA at room temperature. Figure 3 shows that the devices with and without Au nanoparticles exhibited similar spectra peaks at 470 nm and similar full-width half-maximum values of about 18 to 19 nm, demonstrating that the annealing process used to fabricate the Au nanoparticles on the p-GaN layers did not damage the GaN-based LED structure. With an injection current of 100 mA, the EL spectra intensities were enhanced by approximately 55.3% and 41.3% for the Au nanoparticles fabricated from the 2- and 5-nm Au-CNT systems, respectively, compared with the reference conventional planar LEDs. In our EL spectra counting, the peak intensity of LEDs with Au nanoparticles from the 2- and 5-nm Au-CNT systems were 290.8 and 264.6, respectively, compared with 187.2 for conventional LEDs. According to the research status of surface plasmon in LED devices based on GaN materials [12-14,16], the improvement in the optical output power could be attributed to the surface plasmon effect from the Au nanoparticles. Meanwhile, the Au nanoparticles on the surface of LED devices could increase the roughness of the surface. So the enhancement of optical output power may also originate from the surface scattering effect. When comparing the Au nanoparticles from the 5-nm Au-CNT system with the LEDs that had Au nanoparticle arrays from the 2-nm Au-CNT system, the latter showed more enhanced light emission Optical microscopy images of the LEDs with and without the Au nanoparticles with an injection current of 100 mA are shown in the inset of Figure 3. Further optimization of the particle-forming conditions would lead to an even higher increase in the efficiency of the LEDs with nanoparticles from the metal-CNT system in the future.

**Figure 4:**
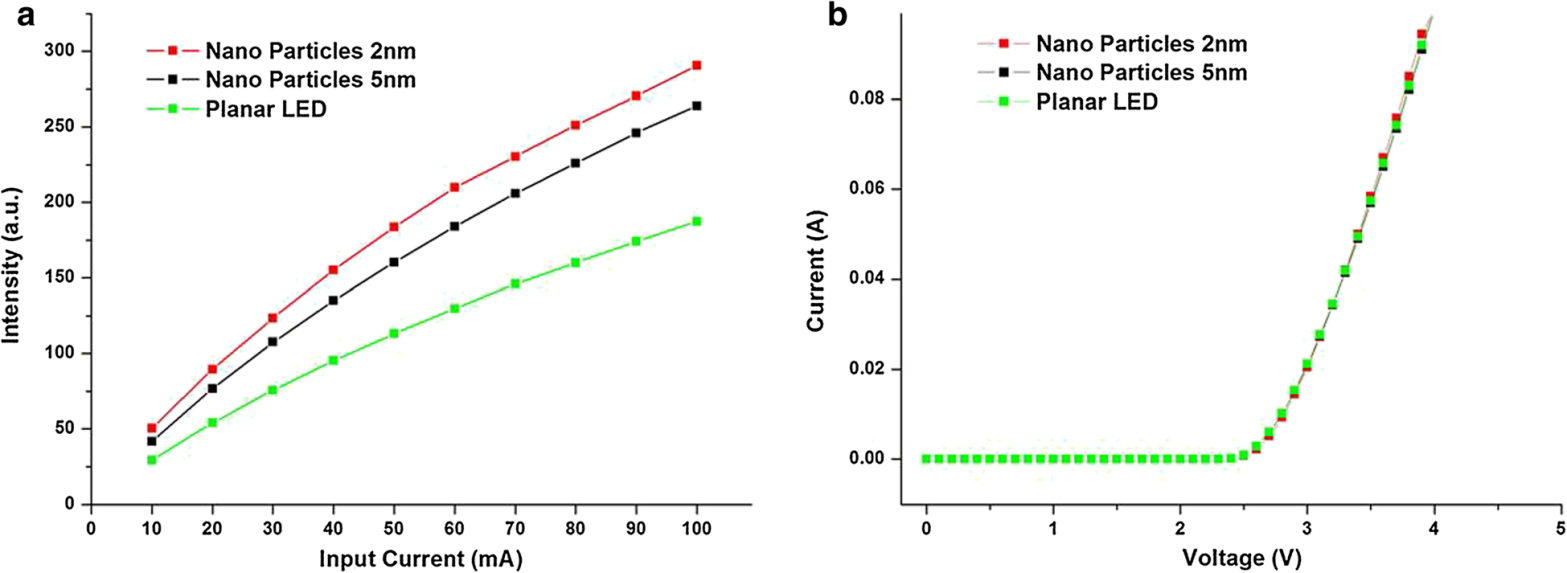
**Optical output power and *I*-*V *characteristics. (a)** Optical output power as a function of the injection current with Au nanoparticles from the 2- and 5-nm Au-CNT systems, compared with a planar LED. **(b)***I*-*V* characteristics of GaN LEDs with Au nanoparticles from the 2- and 5-nm Au-CNT systems compared with a planar LED.

Figure 4a shows the optical output power for the LEDs with and without Au nanoparticles on p-GaN surfaces versus the injection current (L-I) characteristics for all of the devices. The enhancement factor in the optical output power increased as the injection current increased. The voltage–current (*I*-*V*) characteristics for the LEDs with and without an Au nanoparticle layer are shown in Figure 4b. The forward voltage for LEDs with Au nanoparticles on the p-GaN surface was 2.7 V, which is almost the same as that of the planar LEDs without any Au nanoparticles, indicating that fabricating Au nanoparticles on the p-GaN surfaces did not cause the electrical properties to deteriorate.

**Figure 5:**
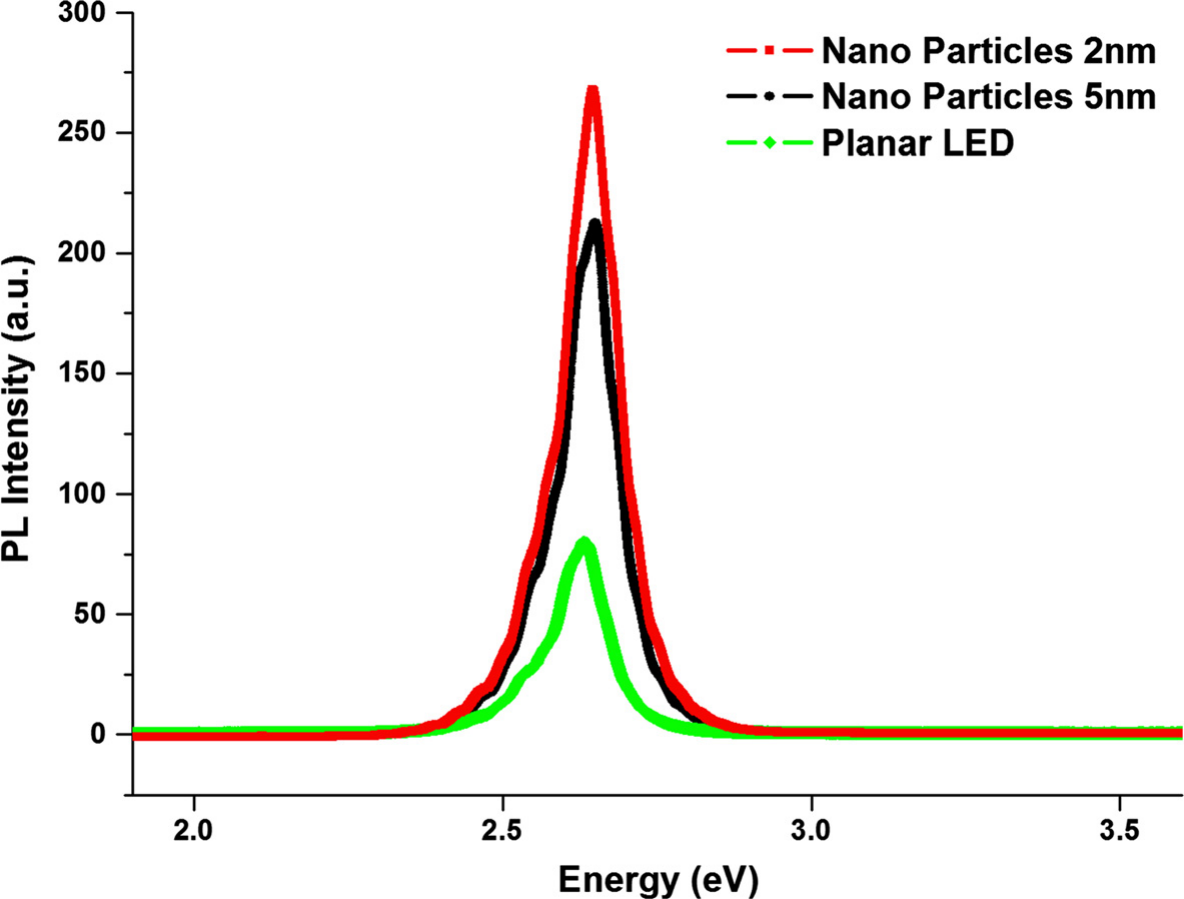
**Room-temperature PL spectra of GaN LEDs.** The LEDs are with Au nanoparticles for the 2- and 5-nm Au-CNT systems with a planar LED as a reference.

To further confirm these results, photoluminescence (PL) spectra measurements were taken for all of the LEDs. The samples were pumped at a normal incidence angle with light from a He-Cd laser source (*λ* = 325 nm) with an excitation laser power of 10 mW at room temperature. The polarization direction of the laser was perpendicular to the Au nanoparticle chains. The laser beam penetrated through an attenuator and then was focused on the sample from the top using a 40 × UV objective lens with a focused spot diameter of approximately 5 μm. Figure 5 shows that the results for the devices from the PL measurements were in agreement with that in the EL experiments. The PL intensity of the LEDs with Au nanoparticles was much higher than that for the planar LEDs. The PL intensity peaks of the LEDs with Au nanoparticles were enhanced by 3.3 and 2.7 times for the 2- and 5-nm Au-CNT systems, respectively.

**Figure 6:**
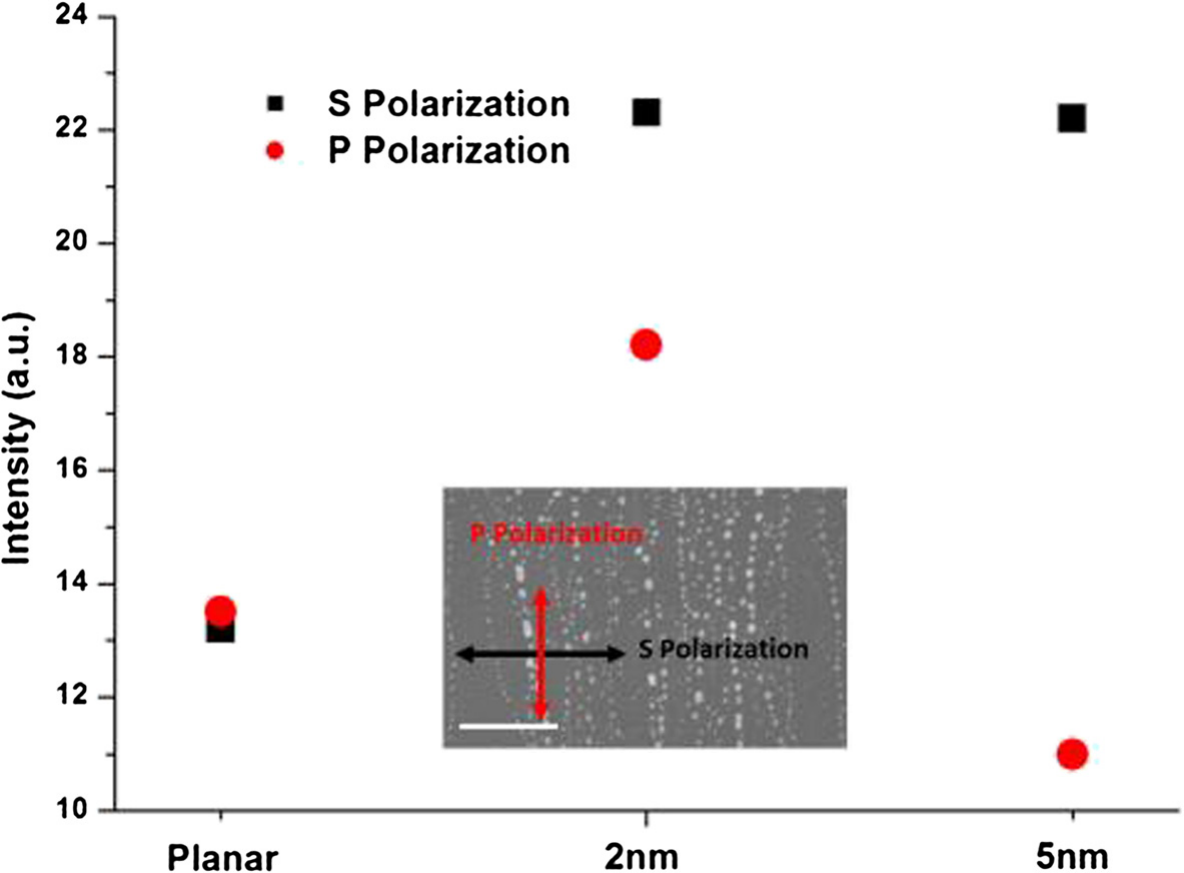
Polarization measurements of LEDs with Au nanoparticles from 2- and 5-nm Au-CNT systems compared with planar LED.

As the Au nanoparticles were distributed along the CNT direction, polarization measurements were performed on the LEDs with Au nanoparticles for the Au-CNT system. Figure 6 shows that the P polarization is defined as the direction that is parallel to the quasi-aligned Au particle array, while the S polarization indicated the vertical direction of the array. There was almost no difference in the intensity between the S and P polarizations with respect to the planar LED, which illustrated that the planar LED was a non-polarized lighting source. For the LEDs with embedded Au nanoparticles derived from the Au-CNT system, polarization was exhibited to a certain degree. The polarization degree was approximately 2.1 and 1.3 for the LEDs with Au nanoparticles derived from the 5- and 2-nm Au-CNT systems, respectively. Compared with the Au nanoparticles derived from the 2-nm Au-CNT system, the 5-nm Au-CNT systems could get Au nanoparticles with a more efficient morphology array for the polarization and a relatively high density. However, the distance between nanoparticle arrays was irregular, and in one nanoparticle array, the space between particles was relatively large in both situations. This gives reason for the unsatisfactory polarization measurements and also provides an effective method in optimizing the Au nanoparticle system.

## Conclusions

In conclusion, the optical output power of the LEDs was enhanced by employing Au nanoparticles fabricated from an Au-CNT system. The enhancement was mainly originated from the surface plasmon effect and surface scattering effect from the Au nanoparticles. The optical output power of these LEDs was enhanced up to 55.3% for an input current of 100 mA. The Au nanoparticle arrays also affected the polarization to a certain degree. Compared with the traditional metal annealing process, Au nanoparticles with a more regular distribution and a controllable size in the subwavelength region could be made using this CNT-based annealing process. This method is simple, cheap, and suitable for mass production in the semiconductor industry.

## Competing interests

The authors declare that they have no competing interests.

## Authors’ contributions

JYH carried out most of the experimental work including all the measurements and drafted the manuscript. LJK prepared the CNT film, and LGH was in charge of metal deposition. CM and ZY carried out the fabrication of LED devices. LQQ conducted the experiment design and analysis of all the experiments, and revised the manuscript as a corresponding author. JKL and FSS participated in all the discussion on this study. All of the authors read and approved the final manuscript.
